# Identification of microRNAs associated with allergic airway disease using a genetically diverse mouse population

**DOI:** 10.1186/s12864-015-1732-9

**Published:** 2015-08-25

**Authors:** Holly Rutledge, Jeanette Baran-Gale, Fernando Pardo-Manuel de Villena, Elissa J. Chesler, Gary A. Churchill, Praveen Sethupathy, Samir N. P. Kelada

**Affiliations:** Department of Genetics, University of North Carolina, 120 Mason Farm Road, Chapel Hill, NC 27599 USA; Curriculum in Bioinformatics and Computational Biology, University of North Carolina, Chapel Hill, NC USA; Curriculum in Genetics and Molecular Biology, University of North Carolina, Chapel Hill, NC USA; Lineberger Comprehensive Cancer Center, University of North Carolina, Chapel Hill, NC USA; The Jackson Laboratory, Bar Harbor, ME USA; Marsico Lung Institute, University of North Carolina, Chapel Hill, NC USA

**Keywords:** Allergic inflammation, Asthma, miRNA, Expression QTL

## Abstract

**Background:**

Allergic airway diseases (AADs) such as asthma are characterized in part by granulocytic airway inflammation. The gene regulatory networks that govern granulocyte recruitment are poorly understood, but evidence is accruing that microRNAs (miRNAs) play an important role. To identify miRNAs that may underlie AADs, we used two complementary approaches that leveraged the genotypic and phenotypic diversity of the Collaborative Cross (CC) mouse population. In the first approach, we sought to identify miRNA expression quantitative trait loci (eQTL) that overlap QTL for AAD-related phenotypes. Specifically, CC founder strains and incipient lines of the CC were sensitized and challenged with house dust mite allergen followed by measurement of granulocyte recruitment to the lung. Total lung RNA was isolated and miRNA was measured using arrays for CC founders and qRT-PCR for incipient CC lines.

**Results:**

Among CC founders, 92 miRNAs were differentially expressed. We measured the expression of 40 of the most highly expressed of these 92 miRNAs in the incipient lines of the CC and identified 18 eQTL corresponding to 14 different miRNAs. Surprisingly, half of these eQTL were distal to the corresponding miRNAs, and even on different chromosomes. One of the largest-effect local miRNA eQTL was for miR-342-3p, for which we identified putative causal variants by bioinformatic analysis of the effects of single nucleotide polymorphisms on RNA structure. None of the miRNA eQTL co-localized with QTL for eosinophil or neutrophil recruitment. In the second approach, we constructed putative miRNA/mRNA regulatory networks and identified three miRNAs (miR-497, miR-351 and miR-31) as candidate master regulators of genes associated with neutrophil recruitment. Analysis of a dataset from human keratinocytes transfected with a miR-31 inhibitor revealed two target genes in common with miR-31 targets correlated with neutrophils, namely *Oxsr1* and *Nsf*.

**Conclusions:**

miRNA expression in the allergically inflamed murine lung is regulated by genetic loci that are smaller in effect size compared to mRNA eQTL and often act in trans. Thus our results indicate that the genetic architecture of miRNA expression is different from mRNA expression. We identified three miRNAs, miR-497, miR-351 and miR-31, that are candidate master regulators of genes associated with neutrophil recruitment. Because miR-31 is expressed in airway epithelia and is predicted to target genes with known links to neutrophilic inflammation, we suggest that miR-31 is a potentially novel regulator of airway inflammation.

**Electronic supplementary material:**

The online version of this article (doi:10.1186/s12864-015-1732-9) contains supplementary material, which is available to authorized users.

## Background

Post-transcriptional control of gene expression is critical for the proper control of inflammation [1]. microRNA (miRNA) mediated transcript degradation and translational inhibition represent two important mechanisms of post-transcriptional control, and a large body of work demonstrates the complex role miRNAs play in fine-tuning the regulatory networks that govern inflammation and innate and adaptive immunity [2]. Allergic airway diseases (AADs) such as allergic rhinitis and asthma are characterized by aberrant adaptive immune responses to allergens that results in airway inflammation, mucus hyper-secretion, and airway hyper-responsiveness [3]. Evidence is accruing that miRNAs play a role in these diseases [4–6]. In particular, several miRNAs have been shown to regulate pathways underlying AAD pathogenesis including T-lymphocyte polarization and function [7–9], regulatory T-cell activity [10], and airway smooth muscle proliferation and contractility [11–13]. Previous studies have also identified miRNAs that are differentially expressed in airway epithelia of individuals with AAD [14–16] and in mouse models of AAD [8, 9, 17]. However, the question of whether genetic variation in miRNA expression affects AAD phenotypes has not been addressed.

We previously characterized the genetics of lung mRNA expression in a mouse model of AAD [18] using the Collaborative Cross (CC) mouse genetics reference population. The CC is a mammalian systems genetics resource comprised of recombinant inbred lines derived from eight-way crosses using five classical inbred strains (C57BL/6 J, 129S1/SvImJ, A/J, NOD/ShiLtJ, and NZO/H1LtJ) and three wild-derived inbred strains (WSB/EiJ, PWK/PhJ, and CAST/EiJ) [19, 20]. Using incipient (i.e., not yet fully inbred) CC lines, which we refer to as “pre-CC” mice, we identified more than 6,000 gene expression quantitative trait loci (eQTL), the majority of which were located near the cognate gene. We used these eQTL to identify candidate genes within QTL for inflammation phenotypes [21]. For example, we identified a locus on chromosome 7 that controls the expression of a gene that is causally related to neutrophil recruitment [22], thereby linking sequence variation, gene expression, and a downstream phenotype. Those analyses were limited to mRNAs. Thus, the role of miRNAs in this experimental system remains unknown.

To identify miRNAs that play a role in AAD, we leveraged the genotypic and phenotypic diversity of preCC mice and utilized two complementary analytical approaches. In the first approach we assessed whether regulatory variation at miRNA loci underlies variation in allergic inflammation. We identified robustly expressed miRNAs that vary by strain, mapped the genetic loci that correlate with expression levels, and then determined whether these loci are associated with AAD phenotypes. In the second approach, we combined bioinformatic sequence analysis with statistical simulations to identify miRNAs that serve as candidate master regulators of genes altered during airway inflammation.

## Methods

Our overall study design and workflow is depicted in Additional file 1: Figure S1.

### Mice

We obtained 129 male preCC mice (ages 10–14 weeks) from Oak Ridge National Laboratory [21, 23, 24]. Each mouse was from an independent CC line that had undergone five to fourteen generations of inbreeding. We also obtained four male mice of each of the eight CC founder strains from The Jackson Laboratory. All mice were singly housed, with alpha-dri bedding, under normal 12-h light/dark cycles. All experiments conducted with mice in this study were compliant with an Institutional Animal Care and Use Committee protocol at an animal facility approved accrediated by the Association for Assessment and Accreditation of Laboratory Animal Care International.

### Phenotyping protocol

We employed a house dust mite (HDM) model of  AAD that produces hallmark disease phenotypes including T_h_2-biased airway inflammation, elevated serum IgE, mucous cell metaplasia, and airway hyper-responsiveness in a strain-dependent fashion [18]. Mice were sensitized with 10 μg of the immunodominant allergen from the *Dermatophagoides pteronyssinus* species of HDM, Der p 1, by intra-peritoneal injection on days 0 and 7, followed by challenge with 50 μg of Der p 1, administered by oro-pharyngeal aspiration, on day 14. On day 17, mice were euthanized, followed by collection of whole lung lavage fluid with two successive volumes of 0.5 and 1.0 ml PBS. No perfusion was performed, so vascular contents were still present in the lungs. Following lavage, lung tissue was snap frozen. Cells in lavage fluid were isolated by centrifugation; eosinophil and neutrophil counts were then manually determined using cytospins and morphologic criteria.

### miRNA expression analysis

For the eight CC founder strains (n = 4/strain), we isolated total lung RNA by Trizol extraction. RNA quality was assessed using an Agilent Bioanalyzer. With one exception, all samples had RNA integrity numbers greater than 7. RNA samples were then processed and hybridized to Affymetrix miRNA 2.0 arrays (GSE63954). We limited our analysis to the 723 probe sets on the array that are specific to mouse miRNAs. Manual inspection of results from principle component analysis of miRNA expression revealed that two samples were outliers (one A/J and one NZO/H1LtJ sample); these two samples were removed prior to subsequent analyses. We used an ANOVA model to identify miRNAs that were differentially expressed by strain at a false discovery rate (FDR) q-value < 0.05. We further filtered this list of miRNAs to those that were highly expressed, which we defined as within the top 20^th^ percentile of miRNA expression in any one CC founder strain, and those that varied by at least 1.5-fold between the highest and lowest-expressing strains. This limited the list to 38 miRNAs. We also selected one miRNA, miR-17* (now known as miR-17-3p), as a reference miRNA for normalization in experiments with preCC mice. miR-17* was selected because its expression was near the mean of all miRNAs that met our selection criteria (mean of all miRNAs = 7.3; mean of miR-17* = 7.1) and it was not differentially expressed by strain (*p* = 0.89).

From these ANOVA models described above, we estimated broad-sense heritability (*H*^2^) by calculating the interclass correlation (r_1_) and the coefficient of genetic determination (g^2^) [25]:

$$ {\mathrm{r}}_1=\left(\mathrm{M}\mathrm{S}\mathrm{B}\hbox{-} \mathrm{M}\mathrm{S}\mathrm{W}\right)/\left(\mathrm{M}\mathrm{S}\mathrm{B}+\left(\mathrm{n}\hbox{-} 1\right)\mathrm{M}\mathrm{S}\mathrm{W}\right) $$

where MSB and MSW are the mean squares between and within, respectively, from the eight-way ANOVA model described above and n is the number of mice per strain. g^2^ is a slightly modified estimate of heritability that accounts for the doubling of the additive genetic variance with inbreeding and is calculated as:

$$ {\mathrm{g}}^2=\left(\mathrm{M}\mathrm{S}\mathrm{B}\hbox{-} \mathrm{M}\mathrm{S}\mathrm{W}\right)/\left(\mathrm{M}\mathrm{S}\mathrm{B}+\left(2\mathrm{n}\hbox{-} 1\right)\mathrm{M}\mathrm{S}\mathrm{W}\right) $$

For the preCC mice, the same RNA isolation protocol was used. We then used Exiqon locked nucleic acid (LNA)-based qRT-PCR assays for each of the 38 miRNAs, except miR-805 (because an assay was not available from Exiqon). We added miR-148b and miR-182 due to published evidence indicating roles in asthma or allergic inflammation in the lung [10, 26], and miR-17* and U6 for normalization. Thus in total we measured the expression of 40 miRNAs and one snRNA (U6). Since the time of ordering the Exiqon LNA qRT-PCR assays, three of the measured miRNAs have had name changes: miR-17* is now miR-17-3p; miR-193* is now miR-193-5p; and miR-322* is now miR-322-3p. We maintained the old nomenclature throughout the manuscript since that is consistent with Exiqon product information at the time of ordering. Exiqon miRCURY LNA Universal RT was used to generate cDNA, after which miRNA-specific primers were used to amplify each miRNA. For each miRNA for each mouse, we calculated the delta Ct relative to miR-17*. Across all samples and miRNAs, the delta Ct relative to miR-17* and U6 snRNA (another commonly used control for normalization) were highly correlated (Pearson r = 0.81, *p* < 5 × 10^−16^). qPCR data is provided as Additional file 2.

### eQTL approach

#### Genotyping and eQTL mapping

We genotyped each mouse at the University of North Carolina at Chapel Hill, using one of two Affymetrix SNP arrays (A or B) that were produced during the development of the Mouse Diversity array (MDA) [27]. After removing uninformative and poorly performing SNPs, these arrays contained 181,752 (A-array) and 180,976 (B-array) SNP assays, and the set of SNPs on each array did not overlap. Most mice (83 %) were genotyped on the B-array and the remaining were genotyped on the A-array. These training arrays were annotated to NCBI Build 36 of the mouse genome, but we mapped QTL boundaries to Build 37 positions to integrate with other resources. We report NCBI Build 37 positions in our results. We estimated the most probable ancestor for each SNP in each mouse using the GAIN algorithm [28], and reconstructed founder haplotypes based on these results. We then merged the non-overlapping SNP datasets from arrays A and B by imputing unobserved genotypes based on inferred founder haplotype. For QTL mapping, we used HAPPY [29] to infer ancestry matrices for an additive genetic model. For computational efficiency, we then averaged the matrices across SNPs between which GAIN inferred no recombination in the population, and this reduced the mapping dataset to 27,059 intervals. Genotype data are provided as Additional file 3. We used BAGPIPE [30] to fit a regression model and calculate LOD scores. Significance thresholds were determined by permutation (*n* = 250 permutations per miRNA), and we used the 1.5 LOD drop method to approximate confidence intervals for QTL [31]. We defined local eQTL as eQTL located within 10 Mb of the cognate gene based on prior work on mRNA eQTL [21]. Narrow sense heritability (*h*^2^) for eQTL was estimated by regression of miRNA expression on CC founder haplotype probabilities. For example, the expression of a given miRNA Y is

$$ \begin{array}{l}\mathrm{Y}\kern0.5em =\kern0.5em \mathrm{i}\mathrm{ntercept}\kern0.5em +\kern0.5em {\mathrm{B}}_1\left(\mathrm{C}57\mathrm{B}\mathrm{L}/6\mathrm{J}\right)\kern0.5em +\kern0.5em {\mathrm{B}}_2\left(129\mathrm{S}1/\mathrm{SvImJ}\right)\kern0.5em +\kern0.5em {\mathrm{B}}_3\left(\mathrm{N}\mathrm{O}\mathrm{D}/\mathrm{ShiLtJ}\right)\kern0.5em +\kern0.5em {\mathrm{B}}_4\left(\mathrm{N}\mathrm{Z}\mathrm{O}/\mathrm{H}1\mathrm{L}\mathrm{t}\mathrm{J}\right)\\ {}+\kern0.5em {\mathrm{B}}_5\left(\mathrm{P}\mathrm{W}\mathrm{K}/\mathrm{P}\mathrm{h}\mathrm{J}\right)\kern0.5em +\kern0.5em {\mathrm{B}}_6\left(\mathrm{C}\mathrm{AST}/\mathrm{E}\mathrm{i}\mathrm{J}\right)\kern0.5em +\kern0.5em {\mathrm{B}}_7\left(\mathrm{W}\mathrm{S}\mathrm{B}/\mathrm{E}\mathrm{i}\mathrm{J}\right)\kern0.5em +\kern0.5em \mathrm{error}\end{array} $$

where each strain term represents the estimated number of that strain’s haplotypes present at the QTL for a given mouse (this estimate being the posterior expectation derived from the probabilistic haplotype reconstruction using HAPPY [29]), and where A/J is designated as the reference strain. We used the model R^2^ to describe the percent of phenotypic variation accounted for by the locus.

### Identification of regions of shared ancestry and phylogenetic analysis

We used a comparative genomic approach to narrow QTL regions and identify candidate genes, as described previously [24]. Using the allele effect plots as a guide, we grouped founder alleles into two groups by effect (*e.g.*, high vs. low allele effect). We divided the two groups based on the greatest difference between ordered allele effects estimated at the QTL peak. We then used the Collaborative Cross Viewer at the University of North Carolina [20, 32] and genome sequences from the Wellcome Trust Sanger mouse genomes project (MGP) [33, 34] to identify regions in the confidence interval in which grouped strains have shared SNP genotypes. Positions identified as heterozygous and low confidence genotype calls were omitted from the analysis.

### Analysis of the effects of SNPs on pre-miRNA structure

To examine the consequences of SNPs on miRNA structure, we used two computational approaches. First, we used RNAfold [35] to compare the predicted structures of pre-miR-342 based on the reference allele (C57BL/6J) to a *musculus*-derived allele (shared by the NOD/ShiLtJ and PWK/PhJ strains) containing two SNPs (rs264778660 and rs242689107) and one indel (rs261236356) that fall within the precursor. Second, we used SNPfold [36] to formally test whether either SNP affects the ensemble of predicted RNA structures.

### Bioinformatic/Statistical approach to identify putative miRNA-mRNA regulatory networks

To construct putative miRNA-mRNA regulatory networks, we utilized a multistep procedure outlined in green in Additional file 1: Figure S1. We first identified mRNA transcripts linearly (positively or negatively) correlated at an FDR < 0.1 with log-transformed eosinophil or neutrophil counts that were previously quantified [21, 22]. We refer to these as called quantitative trait transcripts [37]. Second, we identified quantitative trait miRNAs, which we defined as miRNAs that were linearly correlated with inflammation phenotypes at an FDR < 0.1. For each phenotype (eosinophil or neutrophil counts), we paired positive quantitative trait transcripts with negative quantitative trait miRNAs and vice-versa. We then used a Monte Carlo simulation strategy called miRhub [38, 39] to identify the miRNAs that may act as regulatory hubs for a given set of oppositely correlated quantitative trait transcripts. miRhub identifies miRNAs that are predicted to regulate a target gene list/network more than expected by chance. To accomplish this the algorithm utilizes the following inputs: (1) the strength and clustering of miRNA target sites in the gene set (as predicted by the TargetScan algorithm [40]), these predicted interactions can be filtered to require a minimum level of conservation across species - we specified conservation between mouse and one other species (rat, human, or chicken); (2) the centrality of target genes in a network, which is based on high confidence protein-protein interactions listed in the STRING 9.0 database (http://string-db.org/) - we did not employ this feature; and (3) the expected range of targeting scores in random gene lists/networks (*n* = 1000). Empirical p-values are generated based on the comparison of the observed distribution of target scores to the distribution found in the random gene networks, and are adjusted for multiple comparisons using a false discovery rate.

## Results

As outlined in Additional file 1: Figure S1, we used two complementary approaches to identify miRNAs that may be associated with AAD, one based explicitly on genetically determined variation in miRNA expression (“eQTL approach” shown in blue), and one that does not rely on genetic diversity per se but rather combined bioinformatics and statistical analyses to construct putative miRNA-mRNA regulatory networks (“bioinformatics/statistical approach” shown in green). We begin with results from our survey of miRNA expression in CC founder strains, followed results from the eQTL and bioinformatics/statistical approaches, respectively.

### Detection of differentially expressed miRNAs in the lungs of CC founder strains

**Fig. 1:**
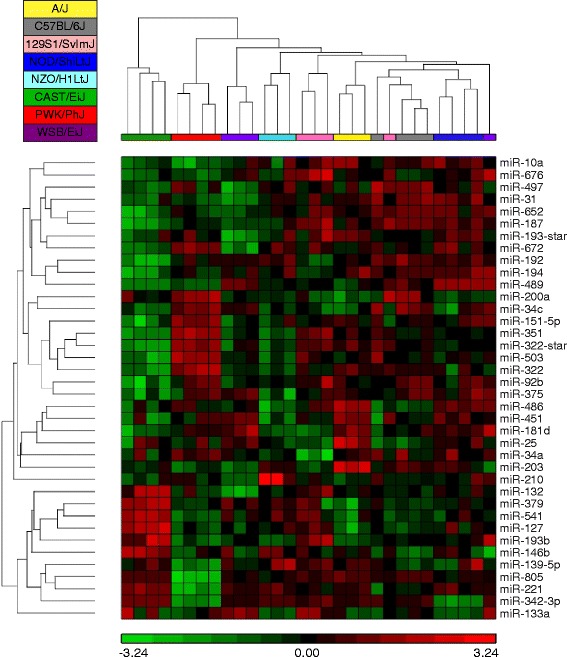
Hierarchical clustering miRNA expression among CC founder lines. Of the 92 differentially expressed miRNAs detected using a microarray platform, 38 were selected based on the expression values and these are depicted here. Note that with one exception (WSB/EiJ mouse at far right), the overall sample clustering is consistent with the phylogenetic relationships among these strains

We measured the expression of 723 miRNAs in whole lung RNA samples from the eight CC founder strains (*n* = 3-4/strain) using microarrays (Methods). We identified 92 that were differentially expressed by strain at an FDR < 0.05 (Additional file 4: Table S1), 38 of which were identified as highly expressed in at least one strain (Methods, Additional file 5: Table S2). Hierarchical clustering of these 38 miRNAs clearly demonstrated the effect of strain (Fig. 1) and the sample clustering was consistent with the known phylogenetic relationships among these strains [41]. We estimated the broad sense heritability (*H*^*2*^) for miRNA expression (Additional file 5: Table S2); *H*^*2*^ values ranged from 0.28 for miR-200a to 0.94 for miR-342-3p.

To ensure that these results were not false positives due to altered hybridization between array probes and genetic variants, we mapped probesets of differentially expressed miRNAs to genetic variants contained in the Sanger Mouse Genomes Project data [33, 34]. Three probesets aligned to regions that contain structural variants among CC founder strains (miR-148b, miR-192, and miR-194), but the observed patterns of expression were not correlated with the strain distribution of structural variants. Thus we conclude that the variation in miRNA expression is not biased by the array platform we used.

### eQTL Approach

We then measured the expression of 37 out of 38 of these miRNAs (Methods), as well as miR-148b and miR-182 due to published evidence indicating roles for these miRNAs in asthma or allergic inflammation in the lung [10, 26], in 129 preCC lines using Exiqon LNA-based qRT-PCR assays. We observed a broad range of expression for many miRNAs (Additional file 1: Figure S2). We note in particular the bimodal distributions for two miRNAs, miR-133 and miR-489, suggesting a single, large-effect eQTL for each.

**Fig. 2:**
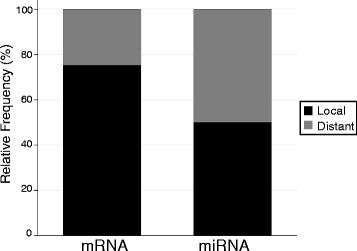
Frequency of mRNA and miRNA eQTL by distance category. Local is defined as within 10 Mb of the gene

Next we performed genome-wide scans for each miRNA to identify miRNA eQTL. In total, we identified 18 high-confidence eQTL (at p_adjusted_ < 0.05) corresponding to 14 different miRNAs, and an additional eight potential eQTL (at p_adjusted_ < 0.10) for seven more miRNAs (Table 1). This equates to high-confidence eQTL for 38 % of the miRNAs that were differentially and highly expressed among the founder strains (and 35 % of the total number of miRNAs studied), though surprisingly miR-133 was not one of them. Of the high-confidence eQTL, nine were located near the miRNA gene itself (within 10 Mb), which we refer to as local eQTL [21], while nine were located distal to the miRNA gene, including on other chromosomes. These results differ from our previous studies of protein coding gene eQTL, which revealed predominately (~75 %) large-effect, local eQTL (Fig. 2) [21].

**Table 1:** miRNA eQTL

miRNA	p value threshold	LOD	Chr	peak (bp)	Start (bp)	End (bp)
miR-139-5p	0.05	7.23	16	75704393	66282944	78490921
**miR-146b**	**0.05**	**10.72**	**19**	**45318029**	**44272094**	**48072092**
**miR-181d**	**0.05**	**12.39**	**8**	**86984259**	**83331794**	**88394118**
**miR-187**	**0.05**	**7.53**	**18**	**24437090**	**17391307**	**25992115**
**miR-203**	**0.05**	**10.20**	**12**	**113443612**	**109075713**	**115064141**
**miR-221**	**0.05**	**7.23**	**X**	**11875846**	**6743622**	**19481467**
miR-25	0.05	7.73	2	29331785	19850261	33013357
miR-322	0.05	8.44	11	111672727	109872770	113662465
**miR-322**	**0.05**	**7.99**	**X**	**49213981**	**46393688**	**72375891**
miR-322*	0.05	7.29	11	111689383	109944727	112970648
**miR-342-3p**	**0.05**	**45.72**	**12**	**109161374**	**108668025**	**110004769**
miR-351	0.05	7.79	7	111699531	104065978	117022949
miR-351	0.05	7.43	X	92256284	72656435	97587740
miR-451	0.05	10.18	9	108231334	106496149	111607827
miR-486	0.05	8.08	9	107619856	105998212	111638630
**miR-489**	**0.05**	**25.31**	**6**	**3360097**	**3183618**	**5399987**
miR-503	0.05	7.39	11	110853290	109531467	112735746
**miR-503**	**0.05**	**7.18**	**X**	**55318092**	**49106938**	**70590355**
miR-148b	0.1	6.66	2	42420095	34807751	50632983
miR-210	0.1	6.62	6	3259542	3001551	13855894
miR-322*	0.1	6.69	7	102716364	86631134	117203425
miR-322*	0.1	6.75	X	55058589	49106938	70017706
miR-34a	0.1	6.73	1	13531834	3036178	30553568
miR-34c	0.1	6.76	13	99709138	94236515	102155480
miR-351	0.1	6.68	11	110853290	106160019	112970648
miR-497	0.1	6.76	11	110106556	106863427	112735746

Bold denotes eQTL located within 10 Mb of the miRNA locus (i.e., local eQTL). Start and end positions (bp) in the Table headings correspond to eQTL confidence intervals

**Fig. 3:**
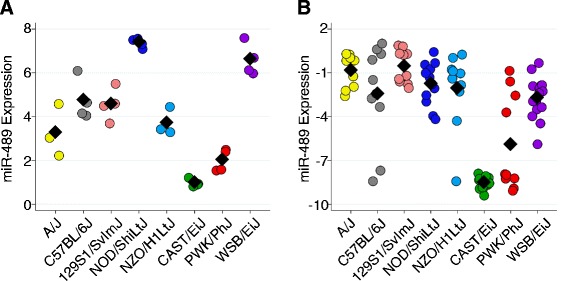
A large effect local eQTL for miR-489. Expression of miR-489 in CC founders (a) and preCC mice (b) as a function of founder haplotype at the eQTL on chromosome 6. CC founder miRNA expression was measured by microarray while preCC miRNA expression was measured by qRT-PCR and data for the latter are presented as −1*DeltaCq (normalized to miR-17*). Black diamonds represent means by strain or haplotype

**Fig. 4:**
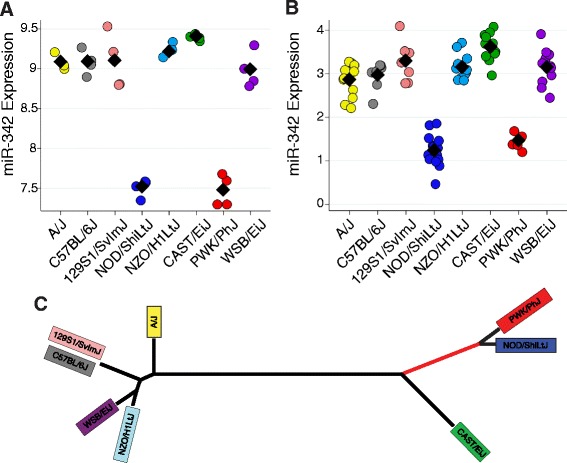
The miR-342-3p eQTL. a. Expression of miR-342-3p in CC founders (a) and preCC mice (b) as a function of founder haplotype at the eQTL on chromosome 12. CC founder miRNA expression was measured by microarray while preCC miRNA expression was measured by qRT-PCR and data for the latter are presented as −1*DeltaCq (normalized to miR-17*). c. Phylogeny of CC founder strains based on SNP data for the region on Chr 12 containing the miR-342-3p eQTL. Bootstrap values were greater than or equal to 96 (out of 100) for each branch of tree.The red line denotes the branch that contains the putative causal variant

We categorized the miRNA eQTL in terms of their effect size and location in the genome. Two eQTL, for miR-489 and miR-342-3p, stood out in terms of effect size. Not surprisingly, expression of these two miRNAs also showed the highest heritability in CC founder lines (Additional file 5: Table S2). We confirmed that for both miRNAs the expression levels among preCC mice with a specific CC founder haplotype at the eQTL were concordant with expression levels in the CC founder strain (Figs. 3 and 4). We calculated the narrow sense heritability (*h*^*2*^) to be 0.60 and 0.84 for the miR-489 and miR-342-3p eQTL, respectively. Taken together, these results indicate that *cis*-regulatory elements at the respective eQTL are the primary determinants of miR-489 and miR-342-3p expression in the lung. However, we note that miR-489 expression in CC founders suggests three distinct groups (high expression group composed of NOD/ShiLtJ and WSB/EiJ; medium expression group composed of A/J, C57BL/6J, 129S1SvImJ, and NZO/H1LtJ; and low expression group composed of CAST/EiJ and PWK/PhJ), but the allele effects for the eQTL on chromosome (Chr) 6 indicates that this eQTL only explains the differences between CAST/EiJ and PWK/PhJ vs. the other six CC founders. Thus it appears that one or more additional loci likely contribute to miR-489 expression; but we did not identify other eQTL (local or distant) using genome scans in which we conditioned on the Chr 6 eQTL.

**Fig. 5:**
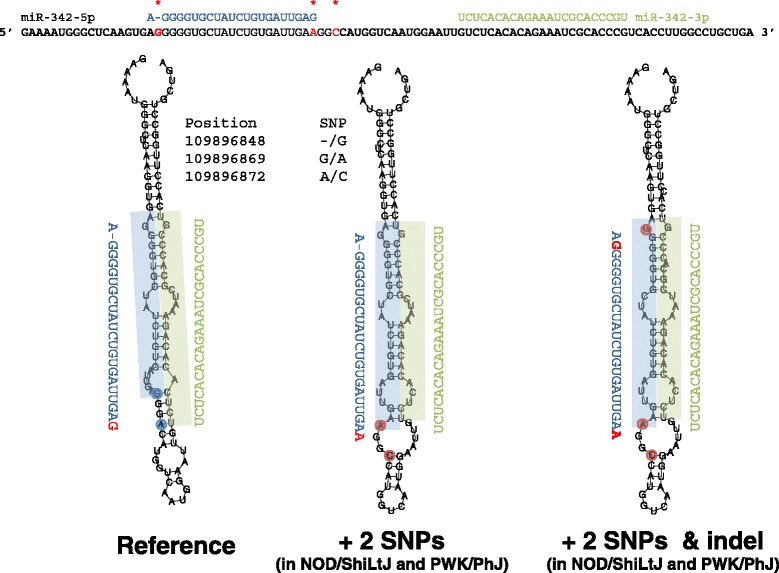
Predicted effects of SNPs in miR-342 on pre-miR-342 structure. Top: sequence spanning 109,896,830-109,896,928 bp on Chr 12 with miR-342-5p and miR-342-3p sequences shown in blue and green, respectively. SNPs present in the *musculus*-derived haplotype are highlighted in red and by asterisks. Bottom: the sequence of miR-342 from C57BL/6J reference strain was used to generate a structure of pre-miR-342, shown on the left. The predicted effect of two SNPs (rs242689107 and rs264778660), present in the *musculus*-derived haplotype, is shown in the center. Note the change in the terminal loop as a function of SNPs. The predicted structure of pre-miR-342 with the addition of the indel (rs261236356) is shown at right

The allele effects for miR-342-3p indicated that the NOD/ShiLtJ and PWK/PhJ alleles were similar in terms of their effect on miRNA expression (Fig. 4b). Using publically available array-based genotype and haplotype data from the CC founder strains [20, 32], we found that, like the *musculus* strain PWK/PhJ, the NOD/ShiLtJ founder strain has a *musculus*-derived haplotype in the QTL confidence interval (Chr 12: 108,668,025-110,004,769 bp). We confirmed that these two strains share a haplotype in this region using more complete single nucleotide polymorphism (SNP) data from the Sanger Mouse Genomes Project [33] (Fig. 4c). This finding facilitated the identification of putative causal variants descended from the *musculus* sub-species that are shared by NOD/ShiLtJ and PWK/PhJ and are different from all other founder strains. We identified 938 SNPs in the region that met these criteria, the majority of which were located in or near 11 genes (defined as +/− 5 kb from the gene), including 20 SNPs in or near the miR-342 locus. Two SNPs (rs264778660 and rs242689107) and one indel (rs261236356) were in the miR-342 precursor (pre-miR-342) and thus were considered strong candidate causal variants for the eQTL because the stem-loop structure of the precursor is critical for miRNA maturation and expression. The RNAfold algorithm [35] predicted an allele-dependent effect for both SNPs (but not the indel) on the terminal loop of the pre-miR-342 stem-loop structure (Fig. 5). Subsequent analysis using SNPfold [36] provided further *in silico* evidence that rs264778660 significantly alters the conformation of pre-miR-342 (*p* = 0.01, Additional file 1: Figure S3). These results suggest that at least rs264778660 is likely to cause a change in pre-miR-342 structure, which in turn may alter processing by Dicer and Drosha, and consequently affect expression of both miR-342-3p and miR-342-5p. Indeed, among CC founder strains, we found a strong correlation between miR-342-3p and miR-342-5p expression, with NOD/ShiLtJ and PWK/PhJ strains having low expression for both arms of miR-342 (Additional file 1: Figure S4). We noted that indel (rs261236356) is predicted to cause a change in the seed sequence of miR-342-5p (Fig. 5), potentially altering the relationship between this miRNA and target genes. We did not measure the expression of miR-342-5p in the preCC because its expression levels were very low.

Seven other miRNAs had local eQTL: miR-146b, miR-181d, miR-187, miR-203, miR-221, miR-322, and miR-503. The allele effects for these eQTL are shown in Additional file 1: Figure S5. Some miRNAs had both local and distant eQTL. For example, miR-322 and miR-503 each had one local eQTL (on Chr X) and one trans-eQTL on Chr 11. We built regression models for these two miRNAs and found that these two loci accounted for 44 % and 37 % of variation in miR-322 and miR-503 expression, respectively.

**Fig. 6:**
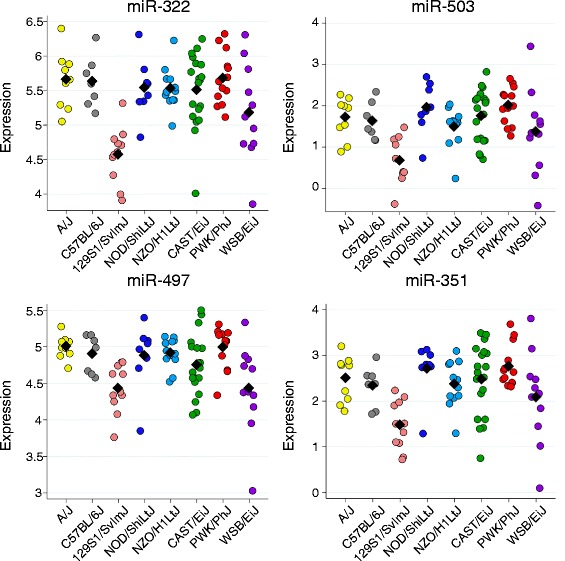
A miRNA trans-eQTL cluster on Chr 11. Allele effects for each miRNA eQTL are shown. Expression derived from the 129S1/SvImJ allele is significantly different from other CC founder strains except WSB/EiJ

The trans-eQTL locus shared by miR-322 and miR-503 was also weakly associated with the expression of miR-351 and miR-497 (p_adjusted_ < 0.1). We found that each pairwise comparison of miRNA expression for all four miRNAs was highly significant (Additional file 6: Table S3) and that the allele effects for all four of these trans-eQTL were consistent (Fig. 6). This suggests that a single variant mediates allele-specific trans-regulation of the expression of all four miRNAs. miRNA expression in mice with the 129S1/SvImJ allele was significantly different from all other strains with the exception of WSB/EiJ (*p* < 0.05 by Tukey’s honestly significant difference test), indicating the 129S1/SvImJ allele is functionally distinct from most other CC founder strains. This region contains 100 genes and we looked for SNPs for which the 129S1/SvImJ strain is distinct from all other CC founders excluding WSB/EiJ and identified 212 SNPs in 20 genes that met this criterion: *A830035A12Rik, Abca6, Amz2, C330019F10Rik, Cacng1, Cacng5, Ccdc46, Gm11650, Gm11655, Gm11674, Gm11677, Gm11678, Gm11680, Gm11681, Gm11714, Gna13, Helz, Prkca, Smurf2,* and *Tex2* (Additional file 7: Table S4)*.* These genes represent causal candidates for the miRNA trans-eQTL. We identified a second set of trans-eQTL on Chr 9 at ~107-108 Mb for miR-486 and miR-451, but the allele effects for these eQTL were not completely consistent (Additional file 1: Figure S6), thus we cannot conclude that these two trans-eQTL are one and the same.

### The relationship between miRNAs and inflammation phenotypes

Genetic variation in a miRNA has been linked to disease phenotypes [42–45]. We asked whether the miRNA eQTL we discovered underlie QTL we previously identified for two phenotypes: eosinophil and neutrophil recruitment responses to allergen sensitization and challenge. No miRNA eQTL co-localized with the eosinophil QTL (Chr 11:71.8-87.1 Mb [21]) or neutrophil QTL (Chr 2:79.8–98.0 Mb and Chr 4:3.3–10.0 Mb [22]).

### Bioinformatic/Statistical approach to identify putative miRNA-mRNA regulatory networks

**Fig. 7:**
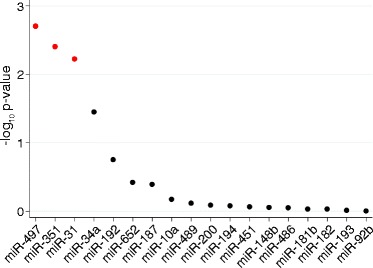
miRNA target site enrichment analysis for neutrophils. Points in red indicate miRNAs with p-values that remain significant after adjusting for multiple testing at an FDR < 0.05

As shown in Additional file 1: Figure S1, we used a complementary approach to ask whether any miRNAs were candidate master regulators of genes altered during airway inflammation. We first identified miRNAs whose expression levels were linearly correlated with eosinophils and neutrophils. At an FDR < 0.1, thirteen and nineteen miRNAs were correlated, respectively. The vast majority was negatively correlated (Additional file 8: Table S5). We then used the miRHub algorithm (Methods), which determines whether the predicted regulatory effect of any given miRNA on a specific set of genes is significantly greater than expected by chance (i.e., acts as a “regulatory hub”), to predict whether any of these miRNAs are candidate drivers of the gene expression profiles associated with the phenotypes of interest. For the set of genes that were positively correlated with neutrophils (*n* = 674 at FDR < 0.1), we identified miR-497, miR-351 and miR-31 as candidate regulatory hubs (Fig. 7). No miRNA hubs were identified for the sets of genes positively (*n* = 1802) or negatively (*n* = 1605) associated with eosinophils (Additional file 1: Figure S7). These results indicate that miR-497, miR-351 and miR-31 may serve as mediators of neutrophilic inflammation by targeting genes that regulate neutrophil recruitment to the airways; predicted targets of each miRNA are listed in Additional file 9: Table S6.

miR-31 has been previously reported as a marker and/or regulator of other inflammatory conditions, such as inflammatory bowel disease [46] and psoriasis [47]. In a previously published dataset of gene expression from human keratinocytes transfected with a miR-31 inhibitor, 96 genes were up-regulated, and of these, 24 were predicted targets of miR-31 [47]. We found that the mouse orthologs of two of these genes, *Oxsr1* and *Nsf*, were also present in our list of predicted miR-31 targets correlated with neutrophils, resulting in statistically significant enrichment (*p* = 4 × 10^−3^ by hyper-geometric test). While an overlap of two genes is not large, it is appreciable given the differences in cell types, species, and disease processes examined. We also found that the expression of *Oxsr1* and *Nsf* was negatively correlated with miR-31 expression (r = −0.26, *p* = 2.0 × 10^−3^ and *r* = −0.23, *p* = 8.7 × 10^−3^, respectively). Thus we conclude that miR-31 is likely to be an important modulator of neutrophilic airway inflammation in part by targeting *Oxsr1* and *Nsf*.

## Discussion

We examined whether regulatory variation at miRNA loci underlies variation in response to allergen sensitization and challenge using a genetically diverse population of mice. The phenotypic variation across these mice enabled the identification of eQTL for 14 out of the 40 miRNAs (35 %) that we studied, as well as suggestive eQTL for an additional seven miRNAs. We characterized these eQTL in terms of effect size and location relative to the miRNA gene and found that we could explain a large fraction of the total variation in expression for several miRNAs using eQTL. Some miRNAs exhibited appreciable heritability estimates based on gene expression in the CC founder strains, but did not have detectable eQTL in the preCC population (e.g., miR-133). One possible explanation is that variation in expression is due to epistasis among two or more loci that lack sizeable individual effects. Due to the limited power afforded by the sample size of our study, we did not test for epistatic eQTL. Additionally, due to the fact that the qRT-PCR system we used was developed by a company and the primer sequences are proprietary, we cannot rule out the possibility that false negative and/or false positive eQTL we detected in the preCC mice are due to SNPs (or other types of genetic variants) that could affect the measurement of miRNA expression.

In the case of miR-342-3p, we were able to identify highly plausible candidate causal variants using a combination of sequence data and structural modeling. We found that a SNP (rs264778660) located in the miRNA precursor is likely to alter the terminal loop of the pre-miR-342 structure, which in turn may alter processing and maturation of miR-342-3p. Liu et al. have shown that the terminal loop of a pre-miRNA affects processing by Dicer [48]. We suggest that rs264778660 affects the maturation and expression of miR-342-3p, providing a plausible explanation for why preCC mice with haplotypes at this locus matching that of NOD/ShiLtJ and PWK/PhJ have lower expression levels. This hypothesis merits further experimental investigation.

Out of all the other miRNAs we studied, very few had variants located in the precursor sequence and none had sequence variants located in seed regions (except for miR-342-5p which was measured in the CC founder lines but not in the preCC population). This is consistent with other population genetic studies, which have concluded that evolutionary pressures have preserved regulatory networks involving miRNAs by selecting against mutations that cause changes in miRNA targeting [49, 50]. 

One of the interesting results from our eQTL analysis was that distal or trans-eQTL were quite common for miRNAs. This contrasts with typical results for mRNA eQTL, including our own findings in the same set of samples [21], where local eQTL constitute the majority. Our findings are similar to those recently reported for miRNA eQTL in human peripheral blood, where miRNA eQTL were often located far upstream from the miRNA itself [51]. In one case, we identified four miRNAs whose expression was regulated by a single locus on Chr 11. Of the ~20 candidate genes in the region, *Prkca*, which encodes Protein Kinase C-alpha, is a plausible candidate because it is a well-known regulator of immune responses in the lung [52] and therefore may regulate pathways that in turn affect miRNA expression.

Trans-eQTL were also more common than local eQTL in a previous miRNA eQTL mapping study involving an F2-intercross with diabetes resistant and susceptible strains (C57BL/6J and BTBR, respectively, each containing two mutant alleles of the leptin gene) [53]. In that study, the authors measured the expression of 220 miRNAs in adipose tissue in 290 F2 mice and identified eQTL for 21 of them (i.e., ~10 % of miRNAs), three of which were local or cis-eQTL. Thus, in comparison, we identified more eQTL (in terms of number of eQTL relative to the number measured) and a larger proportion of local eQTL. It is likely that this can be attributed to the greater genetic diversity present in the CC founder strains versus the two founder strains used in that F2 mapping study. Five miRNAs or miRNA family members were measured in that study and were also a part of this study, namely miR-31, miR-34c, miR-148a, miR-181, and miR-451. We detected a similar local eQTL for miR-181 but there was no overlap in eQTL for the other miRNAs. These differences could be explained at least in part by different regulatory elements responsible for controlling miRNA expression in different tissues (adipose vs. lung) and in response to different perturbations (*ob* transgene/diet vs. allergen). In aggregate, our results and those of Zhao et al. [53] suggest that miRNA expression levels in mice are more strongly dependent on genetic variation at distal loci than mRNAs.

Additionally, the LOD scores for local miRNA eQTL were, on average, lower in magnitude than those for local mRNA eQTL we previously identified. Hence local eQTL account for a smaller proportion of miRNA expression variation than local mRNA eQTL. This too is consistent with results from a recent human miRNA eQTL study in which local eQTL accounted for ~1 % of variation in miRNA expression compared to 30-50 % for mRNA eQTL [51]. Our results indicate that the genetic architecture of miRNA expression is clearly different from mRNA expression, likely involving more loci with additive or perhaps even multiplicative effects. As such, it is perhaps not surprising that we did not identify a miRNA eQTL that underlies variation in allergic inflammation in our model of asthma. For a single miRNA eQTL to underlie a trait QTL, the miRNA eQTL must have a large effect on miRNA expression and be causally linked to the trait. While we identified miRNAs that are correlated with inflammation phenotypes, none of these miRNAs had a single major effect eQTL that explains the resulting variation in inflammation. However, additive or multiplicative effects across miRNA eQTL may explain a greater proportion of the variation in inflammation. More complex statistical models will be required to characterize the effects of multiple loci, and this will inherently require greater statistical power.

The bioinformatic analysis we conducted to identify miRNAs that may act as key regulators of genes involved in granulocyte (eosinophil and neutrophil) recruitment pointed to three miRNAs of interest for neutrophils, namely miR-497, miR-351 and miR-31. Of these, miR-31 is potentially the most promising based on prior work demonstrating that miR-31 is highly expressed in airway epithelia [54], and in skin epithelia regulates the expression of pro-inflammatory chemokines by targeting genes that regulate NF-kB activity [47]. Additionally, miR-31 is not highly expressed in neutrophils at baseline or after bacterial challenge [55], suggesting that miR-31 is not simply a surrogate metric of neutrophil counts. Based on our finding of significant enrichment of predicted miR-31 targets in our data compared to dataset from human keratinocytes in which miR-31 was inhibited [47], we suggest that two genes, *Oxsr1* and *Nsf,* may be novel regulators of neutrophilic inflammation in the airway. NSF (N-ethylmaleimide-sensitive factor) is a cytoplasmic ATPase that plays an important role in the exocytosis of inflammatory mediators by disassembling the soluble-*N*-ethylmaleimide-sensitive-factor accessory-protein receptor (SNARE) complex after exocytosis [56]. Using a mouse model of peritonitis, Morrell et al. showed that inhibition of NSF resulted in diminished neutrophil recruitment to the peritoneum [57]. Given these data, one simple hypothesis is that NSF in airway epithelium regulates the release of a chemokine involved in neutrophil recruitment, and this pathway is targeted by miR-31.

Our results also indicate that other genes with established relationships to neutrophil recruitment are predicted target genes of miR-31. We note three genes in particular: *Ccr2,* a chemokine receptor linked to monocyte and neutrophil recruitment to the lung and other organs [58–60], *Tnip1 (Abin1)*, which is involved in toll-like receptor mediated innate immune responses [61], and *Unc93b1*, a gene involved in antigen presentation by dendritic cells [62]. In combination with prior work in human airway and skin epithelia, our result suggests that miR-31 may be targeting genes both in the airway epithelia and in white blood cells (neutrophils, monocytes, and dendritic cells), and provide rationale for additional studies on the role of miR-31 in neutrophilic airway inflammation. We note that many previous studies have documented an important role for miR-31 in multiple cancer processes [63]; thus our findings suggest that miR-31 is microRNA with pleiotropic effects.

## Conclusion

miRNA expression in the allergically inflamed murine lung is regulated by genetic loci that are smaller in effect size compared to mRNA eQTL and often act in trans. Thus our results indicate that the genetic architecture of miRNA expression is different from mRNA expression. We identified three miRNAs, miR-497, miR-351 and miR-31, that are candidate master regulators of genes associated with neutrophil recruitment. Because miR-31 is expressed in airway epithelia and is predicted to target genes with known links to neutrophilic inflammation, we suggest that miR-31 is a potentially novel regulator of airway inflammation.

## Availability of supporting data

Data for array-based expression of miRNAs in Collaborative Cross founder strains is provided in NCBI’s Gene Expression Omnibus: http://www.ncbi.nlm.nih.gov/geo/query/acc.cgi?acc=GSE63954. Genotype data for preCC mice and qPCR-based measurement of miRNAs in preCC mice are provided as supporting data (Additional Files 2 and 3, respectively).

## Additional files

Additional file 1:
**Figure S1.** Overall study design and workflow. * See Discussion section for a discussion of possible reasons for lack of co-localization. ** See reference 38 for details about this analytical method. **Figure S2.** Distributions of miRNA expression. Data are expressed as Delta Ct vs. U6 snRNA. Note that miR-17* star showed relatively little variation and its mean expression was comparable to the majority of other miRNAs measured; thus it was used as for normalization in subsequent analyses. **Figure S3.** Predicted effects of SNPs in miR-342-3p on miR-342 structure as determined by SNPfold. Green line denotes location of SNP. A. The effect of rs242689107 (G40A) is shown, p = 0.86. B. The effect of rs264778660 (A43C) is significant (p = 0.01). SNP positions refer to miR-342-3p precursor as shown in Fig. 5. **Figure S4.** Expression of miR-342-3p and miR-342-5p in CC founder lines. **Figure S5.** Allele effects for other local miRNA eQTL. **Figure S6.** Allele Effects for miR-451 and miR-486 trans-eQTL on Chr 9. **Figure S7.** miRNA target site enrichment analysis for eosinophils. None of the miRNAs tested had p-values that were significant after adjusting for multiple testing at an FDR < 0.05. Additional file 2:
**qPCR-based measurements of miRNAs in preCC mice.**
Additional file 3:
**Genotype data for preCC mice.**
Additional file 4: Table S1. Differentially expressed miRNAs among CC founder strains. Additional file 5: Table S2. miRNA expression and heritability among CC founder strains for select miRNAs. Additional file 6: Table S3. Pairwise Pearson Correlation Values Among miR-322, miR-252, miR-497, and miR-503. Additional file 7: Table S4. SNPs on Chromosome 11 in the miRNA trans-band eQTL interval for which the 129S1/SvImJ Strain is distinct. Additional file 8: Table S5. miRNAs correlated with inflammation phenotypes. Additional file 9: Table S6. Predicted target genes of enriched miRNAs.

## Abbreviations

AAD: allergic airway disease
CC: Collaborative Cross
eQTL: Expression QTL
HDM: house dust mite
preCC: Pre-Collaborative Cross
QTL: Quantitative trait locus/loci
