# Supplementary material for: Identification of microRNAs associated with allergic airway disease using a genetically diverse mouse population
Source: BMC Genomics. 2015 Aug 25;16(1):633. doi: 10.1186/s12864-015-1732-9 (PMC4548451; doi:10.1186/s12864-015-1732-9)
Supplement: Additional file 1: — Figure S1. Overall study design and workflow. * See Discussion section for a discussion of possible reasons for lack of co-localization. ** See reference 38 for details about this analytical method. Figure S2. Distributions of miRNA expression. Data are expressed as Delta Ct vs. U6 snRNA. Note that miR-17* star showed relatively little variation and its mean expression was comparable to the majority of other miRNAs measured; thus it was used as for normalization in subsequent analyses. Figure S3. Predicted effects of SNPs in miR-342-3p on miR-342 structure as determined by SNPfold. Green line denotes location of SNP. A. The effect of rs242689107 (G40A) is shown, p = 0.86. B. The effect of rs264778660 (A43C) is significant (p = 0.01). SNP positions refer to miR-342-3p precursor as shown in Fig. 5. Figure S4. Expression of miR-342-3p and miR-342-5p in CC founder lines. Figure S5. Allele effects for other local miRNA eQTL. Figure S6. Allele Effects for miR-451 and miR-486 trans-eQTL on Chr 9. Figure S7. miRNA target site enrichment analysis for eosinophils. None of the miRNAs tested had p-values that were significant after adjusting for multiple testing at an FDR < 0.05. [file 12864_2015_1732_MOESM1_ESM.pptx]

## Slide 1
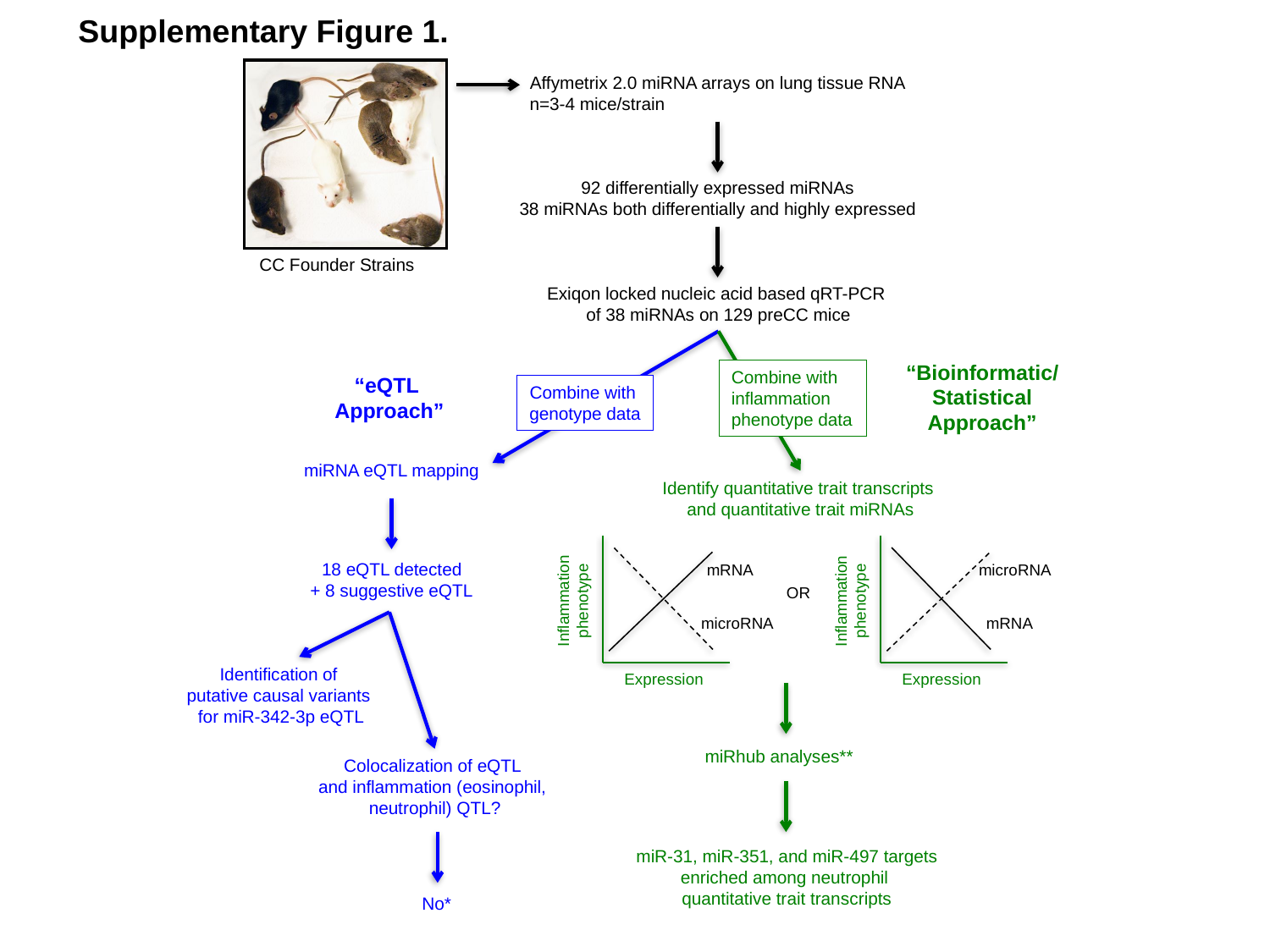

Supplementary Figure 1.
Affymetrix 2.0 miRNA arrays on lung tissue RNA
n=3-4 mice/strain
92 differentially expressed miRNAs
38 miRNAs both differentially and highly expressed
CC Founder Strains
Exiqon locked nucleic acid based qRT-PCR
of 38 miRNAs on 129 preCC mice
“Bioinformatic/
Statistical
Approach”
Combine with inflammation
phenotype data
“eQTL
Approach”
Combine with
genotype data
miRNA eQTL mapping
Identify quantitative trait transcripts
and quantitative trait miRNAs
mRNA
Inflammation
phenotype
microRNA
Expression
microRNA
Inflammation
phenotype
mRNA
Expression
18 eQTL detected
+ 8 suggestive eQTL
OR
Identification of
putative causal variants
for miR-342-3p eQTL
miRhub analyses**
Colocalization of eQTL
and inflammation (eosinophil,
neutrophil) QTL?
miR-31, miR-351, and miR-497 targets
enriched among neutrophil
quantitative trait transcripts
No*

## Slide 2
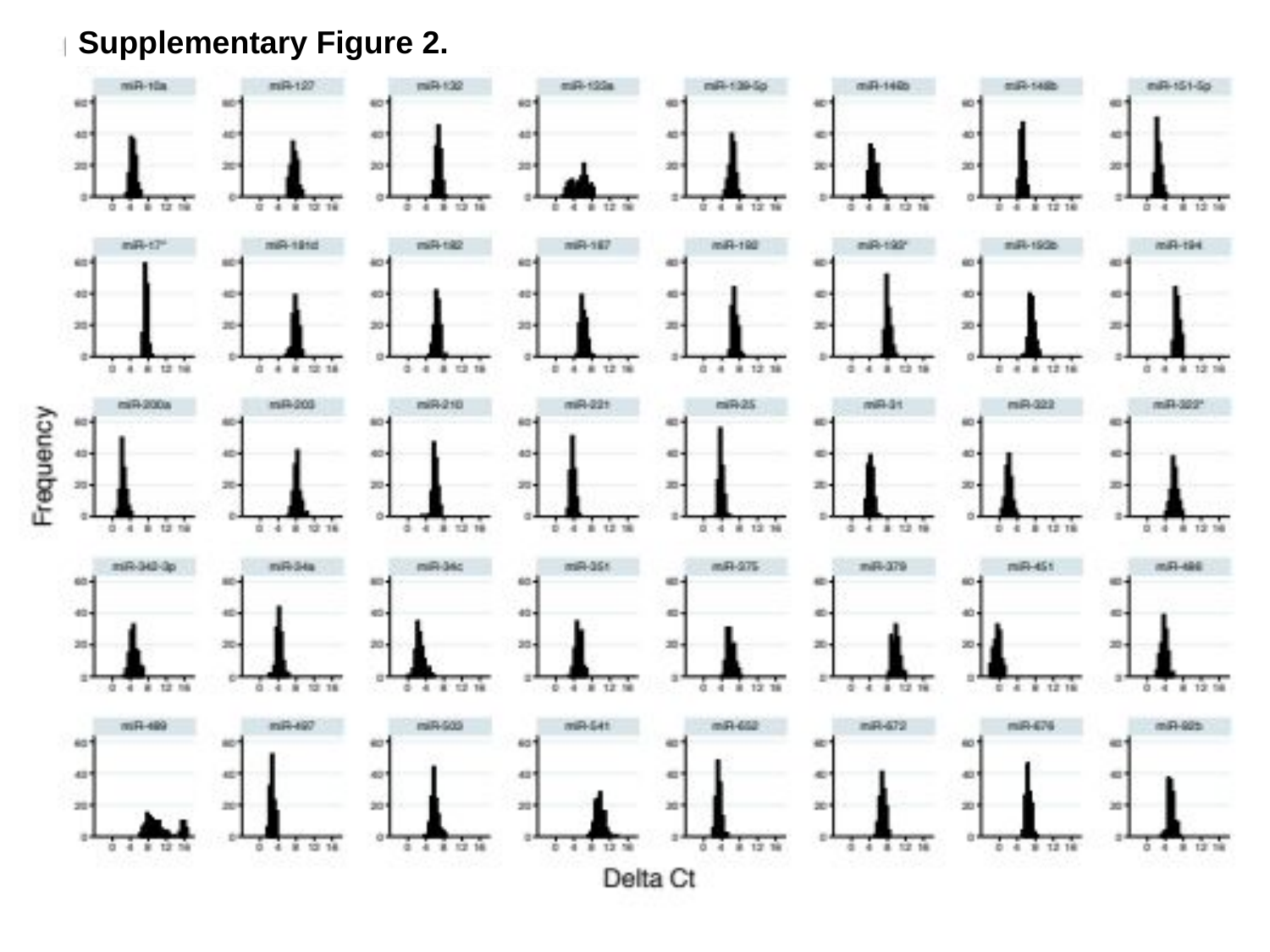

Supplementary Figure 2.
#

## Slide 3
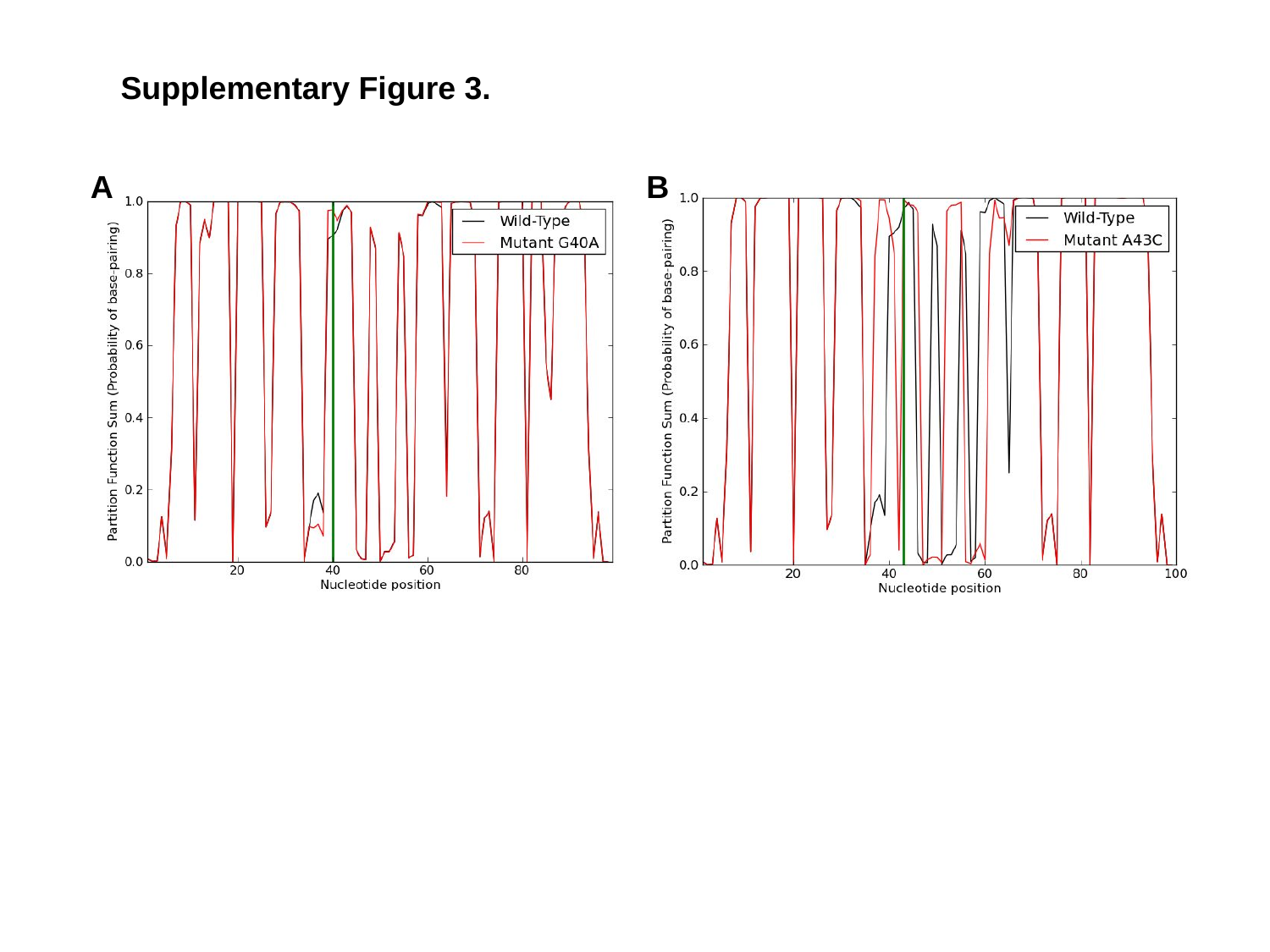

Supplementary Figure 3.
A
B

## Slide 4
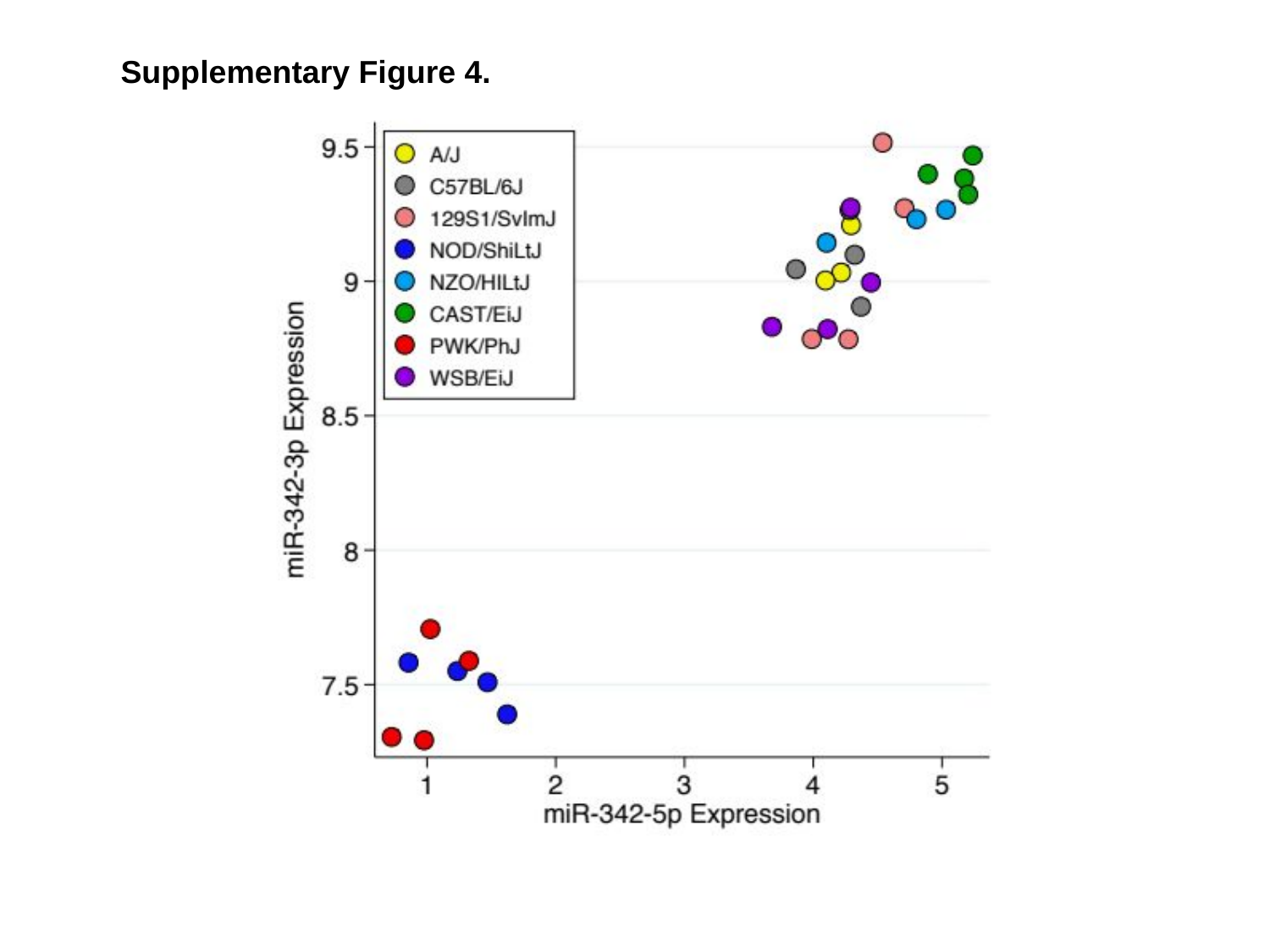

Supplementary Figure 4.

## Slide 5
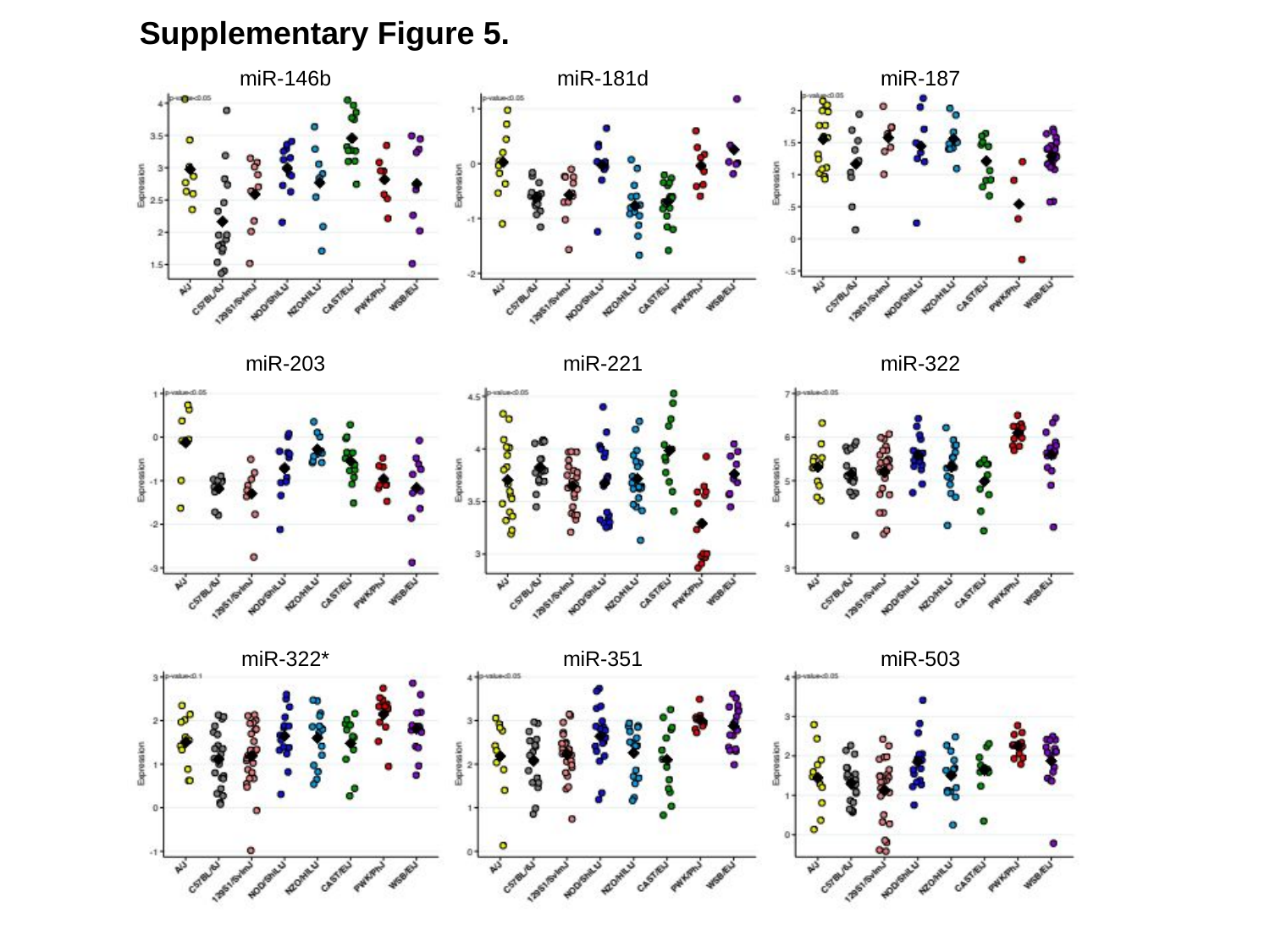

Supplementary Figure 5.
miR-146b
miR-181d
miR-187
miR-203
miR-221
miR-322
miR-322*
miR-351
miR-503

## Slide 6
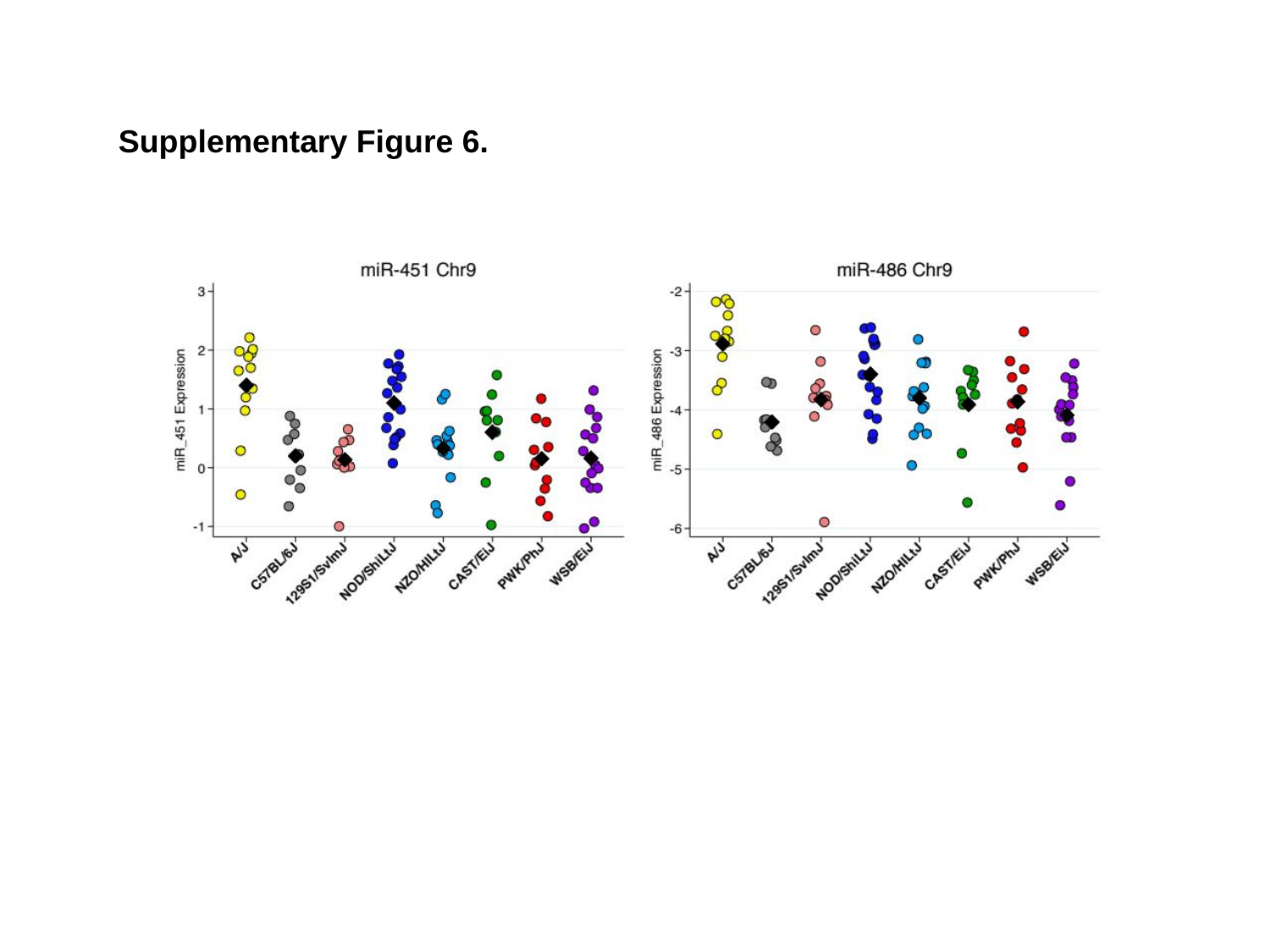

Supplementary Figure 6.

## Slide 7
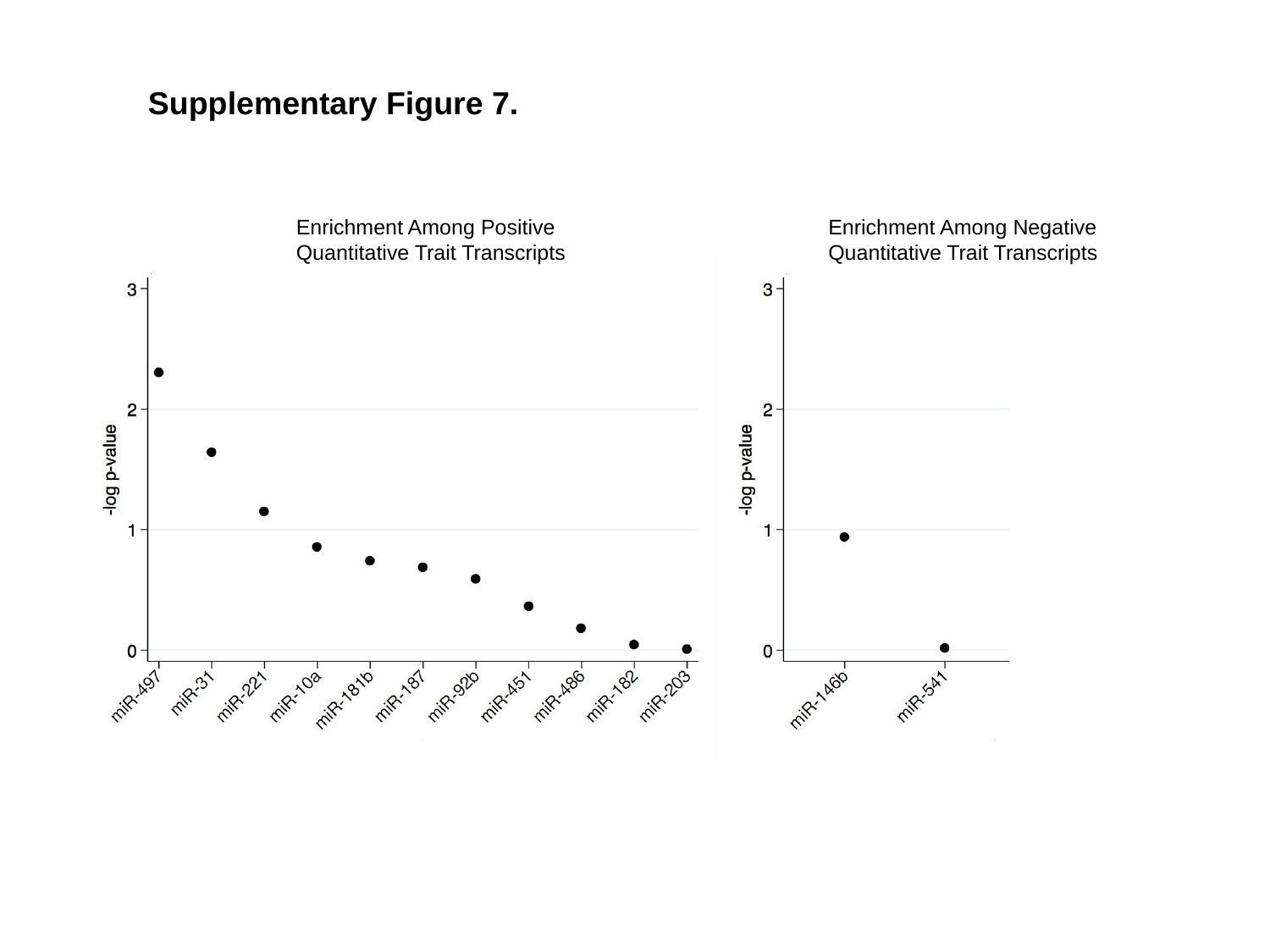

Supplementary Figure 7.
Enrichment Among Positive
Quantitative Trait Transcripts
Enrichment Among Negative
Quantitative Trait Transcripts
