# Supplementary material for: Identification of microRNAs associated with allergic airway disease using a genetically diverse mouse population
Source: BMC Genomics. 2015 Aug 25;16(1):633. doi: 10.1186/s12864-015-1732-9 (PMC4548451; doi:10.1186/s12864-015-1732-9)
Supplement: Additional file 6: Table S3. — Pairwise Pearson Correlation Values Among miR-322, miR-252, miR-497, and miR-503. [file 12864_2015_1732_MOESM6_ESM.docx]

Table S3. Pairwise Pearson Correlation Values Among miR-322, miR-252, miR-497, and miR-503

|  | miR-322 | miR-351 | miR-497 | miR-503 |
| --- | --- | --- | --- | --- |
| miR-322 | 1.00 |  |  |  |
| miR-351 | 0.74 | 1.00 |  |  |
| miR-497 | 0.61 | 0.45 | 1.00 |  |
| miR-503 | 0.81 | 0.74 | 0.44 | 1.00 |

All values are significant at p<1x10^-4^.
